# Simulation of immiscible liquid–liquid flows in complex microchannel geometries using a front-tracking scheme

**DOI:** 10.1007/s10404-018-2149-y

**Published:** 2018-10-25

**Authors:** Lyes Kahouadji, Emilia Nowak, Nina Kovalchuk, Jalel Chergui, Damir Juric, Seungwon Shin, Mark J. H. Simmons, Richard V. Craster, Omar K. Matar

**Affiliations:** 1 Department of Chemical Engineering, Imperial College London, South Kensington Campus, London, SW7 2AZ UK; 2 School of Chemical Engineering, University of Birmingham, Birmingham, B15 2TT UK; 3 College of Sciences, Massey University, Auckland, 0745 New Zealand; 4 Laboratoire d’Informatique pour la Mécanique et les Sciences de l’Ingénieur (LIMSI), Centre National de la Recherche Scientifique (CNRS), Université Paris Saclay, Bât. 507, Rue du Belvédère, Campus Universitaire, 91405 Orsay, France; 5 Department of Mechanical and System Design Engineering, Hongik University, Seoul, 121-791 South Korea; 6 Department of Mathematics, Imperial College London, South Kensington Campus, London, SW7 2AZ UK

**Keywords:** Two-phase flow, Cross-junction, Pancakes, Plugs, Drops, Jets

## Abstract

The three-dimensional two-phase flow dynamics inside a microfluidic device of complex geometry is simulated using a parallel, hybrid front-tracking/level-set solver. The numerical framework employed circumvents numerous meshing issues normally associated with constructing complex geometries within typical computational fluid dynamics packages. The device considered in the present work is constructed via a module that defines solid objects by means of a static distance function. The construction combines primitive objects, such as a cylinder, a plane, and a torus, for instance, using simple geometrical operations. The numerical solutions predicted encompass dripping and jetting, and transitions in flow patterns are observed featuring the formation of drops, ‘pancakes’, plugs, and jets, over a wide range of flow rate ratios. We demonstrate the fact that vortex formation accompanies the development of certain flow patterns, and elucidate its role in their underlying mechanisms. Experimental visualisation with a high-speed imaging are also carried out. The numerical predictions are in excellent agreement with the experimental data.

**Electronic supplementary material:**

The online version of this article (doi:10.1007/s10404-018-2149-y) contains supplementary material, which is available to authorized users.

## Introduction

**Fig. 1:**
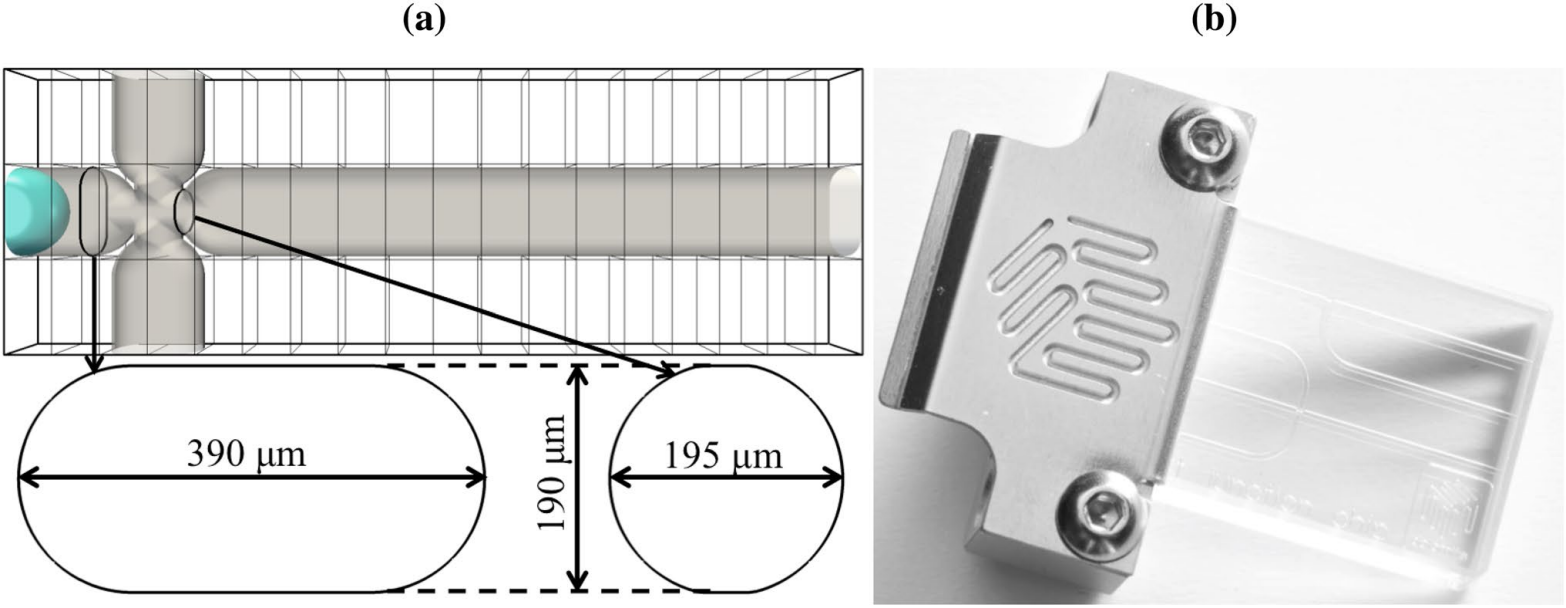
Configuration of Dolomite droplet junction chip with etch depth $190\,\upmu \hbox {m}$ (part number 3000301) [7] used for this study **a**; numerical design and calculation domain of size 3.42 mm length, 0.19 mm width and 1.14 mm height **b**. The calculation domain is divided into $18 \times 1 \times 3 = 54$ subdomains, as shown in **a**, where each subdomain holds a regular grid mesh of $32\times 32 \times 64$ cells. The global resolution in the entire domain is then $576\times 32\times 192$

Two-phase flow in microchannels is of central importance to applications in chemical, medical, and pharmaceutical processes such as inkjet printing, DNA chips, lab-on-a-chip technology, micro-propulsion, and microfluidics (Andersson and Berg 2003; Kuswandi et al. 2007; Martinez et al. 2010; Squires and Quake 2005; Mark et al. 2010). Many configurations (Christopher and Anna 2007) are used in applications involving co-flowing and cross-flowing streams, flow in an elongation channel, and stretching-dominated flows for which droplet, or plug formation is obtained by a periodic breakup mechanism of the dispersed phase. The most popular device designs, however, are flow-focusing (Sugiura et al. 2004; Yasuno et al. 2004), T-junctions (Garstecki et al. 2006; Graaf et al. 2006; Menech et al. 2008), and cross-junctions (Liu and Zhang 2011).

Experimental studies have highlighted the importance of the squeezing mechanism on the droplet, or plug, at the junction by providing the plug size as a function of flow-rate ratio fitted by a simple scaling law. Garstecki et al. (2006), Thorsen et al. (2001), Tice et al. (2004) and Christopher et al. (2008) amongst others all studied these squeezing regimes for the case of a square section T-junction channel; Guillot et al. Guillot and Colin (2005) provided similar studies for both square and rectangle cross-section of T-junction channel. Naturally, many devices do not involve simple square cross-section channels, and the details of the junction often involve additional detail and narrowing to fine-tune the breakup by further intensifying the flow at the junction. The influence of the device cross-section, junction-thinning, and details of the breakup itself have not been the subject of a thorough numerical study; this is the aim of the present paper. The lattice Boltzmann method (Succi 2001) has been used for multiphase microchannel devices with square (Liu and Zhang 2011) or rectangle (Menech et al. 2008) cross-sections for T-junction configurations. Other techniques have also been used to conduct numerical simulations of microfluidic flows, such as the volume-of-fluid, and level-set methods, though the latter feature numerical instabilities particularly when the interfacial tension becomes a dominant factor in the flow (Shyy et al. 1996).

The front-tracking technique (Unverdi and Tryggvason 1992), and the variants developed by Shin and Juric (2009a, b) and Shin et al. (2017, 2018), exhibit no numerical instabilities, and parasitic currents. This approach is ideally suited to multiphase flow simulation, particularly in the case of surface tension-dominated flows, and it is employed herein to study the physics of breakup, the influence of the flow-focussing at junctions in microfluidics devices, which are potentially key, as shown in previous experimental work. For instance, Steijn et al. (2009) performed experiments for rectangular T-junctions and provided important details of the flow just before the thread pinch-off, highlighting the existence of a reverse flow, around the thread, just when the neck collapses rapidly to release a drop. More recently, Chinaud et al. (2015) have developed a new technique for flow visualisation, termed “complementary micro Particle Image Velocimetry ($\mu PIV$)”, which allows velocity fields in both phases to be imaged. These experiments highlight the apparent existence of an intriguing vortex formation during the squeezing regime; we utilise the results of our simulation technique to detect, and quantify numerically, the role of this vortex in the breakup mechanism.

The rest of this article is organised as follows: the channel geometry construction is covered in Sect. 2, while the governing equations, the computational methods, and the problem initialisation are outlined in Sect. 3 together with a description of the numerical techniques for interface advection. Sect. 4 presents results and flow details for two types of squeezing regimes together with cross-validation results against experimental data. Finally, concluding remarks are provided in Sect. 5.

## The configuration of the cross-junction and its numerical construction 

The specific micro-channel device used in this study is similar to the device used by Kovalchuk et al. (2018), illustrated in Fig. 1; here, the geometry acts to both focus flow and combine different fluids using a cross-junction. This glass microfluidics device is designed by Dolomite as a droplet junction chip, with etch depth 190 $\upmu \hbox {m}$ (part number 3000301) [7]. The complexity of the cross-junction is not simply in terms of its plan-form, but also in its cross-section with the channels having non-circular, and different, tubular forms; at the junction itself, there is designed constriction. Our approach circumvents the need for time-consuming construction, meshing, and remeshing, of this geometry. Instead, we proceed in a modular manner that enables us to build the geometry from primitive geometrical objects using a static distance function that takes into account the interaction of these objects with the flow for both single and two-phase flows. The final structure in the computational domain, viz. Figure 1-top-left, consists of the iso-value $\psi (x,y,z) =0$; the static distance function, $\psi (x,y,z)$, is positive for the fluid part and negative for the solid part, and (*x*, *y*, *z*) are Cartesian coordinates.

**Fig. 2:**
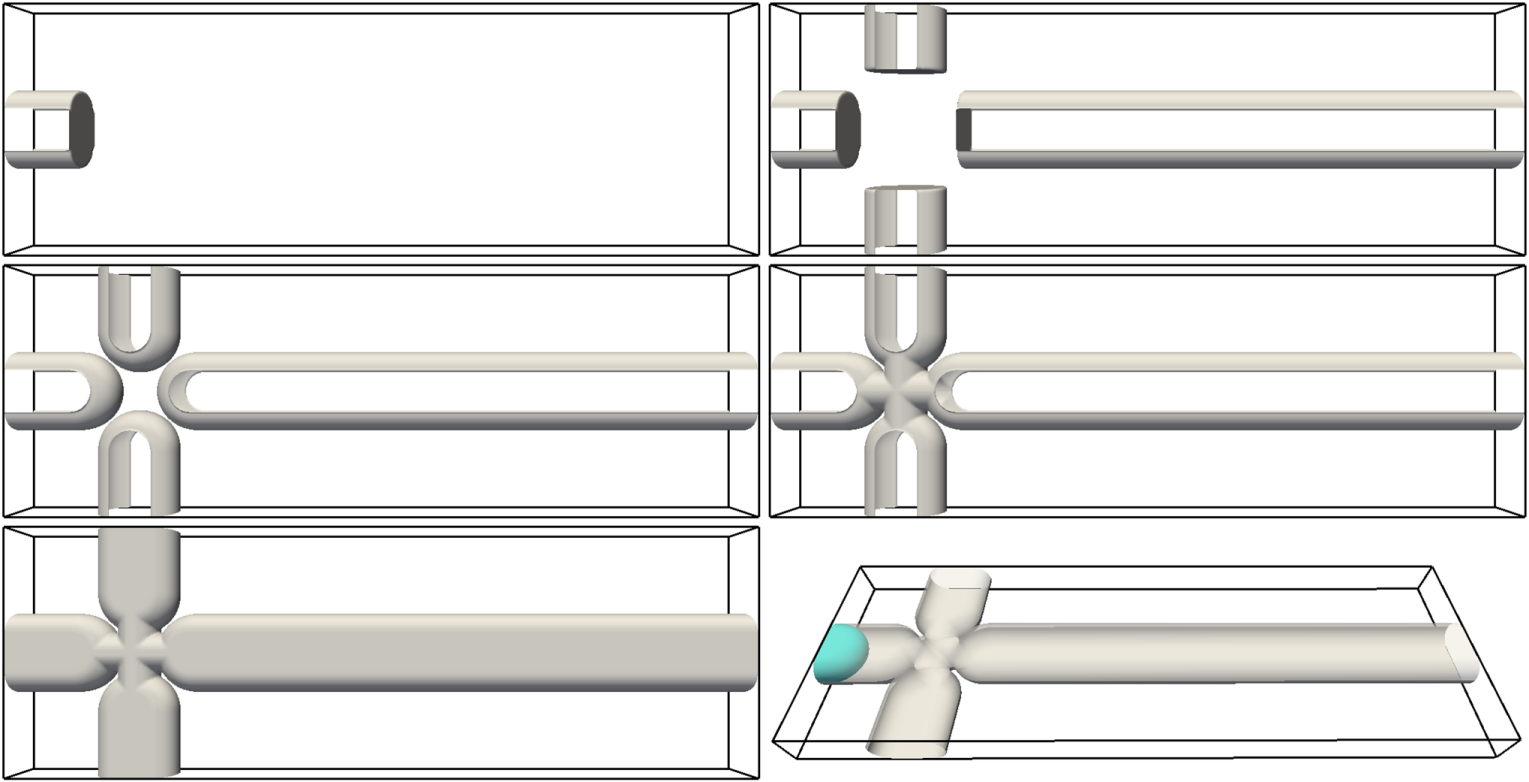
The modular construction of the geometry as it is built from left to right and top to bottom

Many primitive solid geometry shapes are already included in the code, including spheres, planes, cylinders, and tori, as are geometrical operations such as the union or intersection for each primitive object. In our case, only planes, cylinders and tori are required for the construction of the cross-junction and they are easily combined together and Fig. 2 illustrates the steps of the construction:

- Starting with one of the branches, here the left side requires an intersection of two horizontal planes at a distance of $200\,\upmu \mathrm{m}$, followed by a union with two horizontal cylinders with a diameter of $190\,\upmu \mathrm{m}$, and, finally, an intersection with a perpendicular plane in the spanwise direction (viz. Fig. 2, top-left).
- The left branch is then stored, and in a similar way we construct and store the right, top, and bottom branches. We then assemble all the branches using a union operation (Fig. 2 top-right).
- An oval structure is added at the end of each branch: this is the union of a torus and cylinder, as shown in Fig. 2, center-left.
- The junction is made by the union of two perpendicular cylinders of a diameter of $195\,\upmu \mathrm{m}$. We use the union operation to combine this with the branches (viz. Fig. 2, center-right).
- Finally, we close the resulting channel intersecting two planes in the front and the back of the domain (Fig. 2, bottom-left).

Similar techniques are used for the construction of the initial shape of the fluid interface. The inlet section has an oval shape (see Fig. 1, bottom-left), and the interface is initialised with the shape of a half-pancake at the inlet (see Fig. 2, bottom-right). This pancake shape is a union of a torus and a cylinder, and the intersection of two planes.

## Problem formulation

### Governing equations

We now outline the basic solution procedure for the Navier–Stokes equations together with a brief explanation of the interface method; full details of the numerical solution method for the velocity, pressure and interface dynamics are given in several articles of Shin and Juric Shin et al. (2005), Shin and Juric (2007), Shin (2007) and Shin and Juric (2009a, b). The governing equations for transport of an incompressible two-phase flow are given as:

1 $$\begin{aligned} \nabla \cdot \mathbf{u }&= 0 \nonumber \\ \rho \left( \frac{\partial \mathbf{u }}{\partial t} +\mathbf{u }\cdot \nabla \mathbf{u }\right)&= -\nabla P + \nabla \cdot \mu \left( \nabla \mathbf{u } +\nabla \mathbf{u }^T \right) + \mathbf{F } \end{aligned}$$

where $\mathbf{u }$ is the velocity, *P* the pressure and $\mathbf{F }$ is the local surface tension force at the interface. This force is described by the hybrid formulation:

2 $$\begin{aligned} \mathbf{F } = \sigma \kappa _{_H}\nabla \textit{I} \end{aligned}$$

where $\sigma$ is the surface tension coefficient assumed here to be constant. We use an indicator function, $\textit{I}$ , that is zero in one phase and one in the other phase. Numerically this transition is resolved across three-to-four grid cells with a steep but smooth numerical Heaviside function generated using a vector distance function computed directly from the tracked interface (Shin and Juric 2009a). A curvature function, $\kappa _{_H}$, is defined to be twice the mean interface curvature field and it is calculated on the Eulerian grid using:

3 $$\begin{aligned} \kappa _{_H} = \frac{\mathbf{F }_{_L}\cdot \mathbf{G }}{\sigma \mathbf{G }\cdot \mathbf{G }} \end{aligned}$$

4 $$\begin{aligned} \text{ where }\,\,\,\, \mathbf{F }_{_L}= & \int _{\varGamma (t)} \sigma \kappa _{_f} \mathbf{n }_{_f} \delta _{_f} \left( \mathbf{x } - \mathbf{x }_{_f} \right) \hbox {d}s \end{aligned}$$

5 $$\begin{aligned} \text{ and }\,\,\,\, \mathbf{G }= & \int _{\varGamma (t)} \mathbf{n }_{_f} \delta _{_f} \left( \mathbf{x } - \mathbf{x }_{_f} \right) \hbox {d}s. \end{aligned}$$

In these formulae: $\mathbf{x }_{_f}$ is a parameterisation of the interface, $\varGamma (t)$, and $\delta _{_f}(\mathbf{x }-\mathbf{x }_{_f})$ is a Dirac distribution that is non-zero only when $\mathbf{x }=\mathbf{x }_{_f}$, $\mathbf{n }_{_f}$ is the unit normal vector to the interface and $\hbox {d}s$ is the length of the interface element; $\kappa _{_f}$ is again twice the mean interface curvature but now obtained from the Lagrangian interface structure. The geometric information, unit normal, $\mathbf{n }_{_f}$, and length of the interface element, $\text{ ds }$, in $\mathbf{G }$ are computed directly from the Lagrangian interface and then distributed onto an Eulerian grid using the discrete delta function. The details follow Peskin’s (1977) well-known immersed boundary approach and a description of our procedure for calculating the force and constructing the function field $\mathbf{G }$ and indicator function $\textit{I}$ is given in Shin et al. (2005), Shin and Juric (2007), Shin (2007), Shin and Juric (2009a, b). The Lagrangian interface is advected by integrating

6 $$\begin{aligned} \frac{\hbox {d}\mathbf{x }_{_f}}{\text{ dt }} = \mathbf{V } \end{aligned}$$

with a second-order Runge–Kutta method where the interface velocity, $\mathbf{V }$, is interpolated from the Eulerian velocity. The continuous phase is subject to the no-slip condition at the solid boundaries. The dispersed phase in the examples presented here does not interact with the solid boundaries but in the general case, were contact to occur, the triple line contact model described in Shin and Juric (2009b) and Shin et al. (2018) is used. Material properties, such as the density and viscosity, are defined in the entire domain with the aid of the indicator function $\textit{I}(\mathbf{x },t)$:

7 $$\begin{aligned} \left. \begin{array}{c} \rho (\mathbf{x },t) = \rho _{_\mathrm{con.}} +\left( \rho _{_\mathrm{dis.}} -\rho _{_\mathrm{con.}}\right) \textit{I}(\mathbf{x },t)\\ \mu (\mathbf{x },t) = \mu _{_\mathrm{con.}} +\left( \mu _{_\mathrm{dis.}} -\mu _{_\mathrm{con.}}\right) \textit{I}(\mathbf{x },t) \end{array}\right. \end{aligned}$$

where the subscripts $_\mathrm{con.}$, and $_\mathrm{dis.}$, stand for the continuous and dispersed phases, respectively. The numerical code structure consists of two main modules:

- A module that solves the incompressible Navier–Stokes equations
- A module for the interface solution that includes tracking the phase front, initialisation and reconstruction of the interface when necessary.

The parallelization of the code is based on an algebraic domain-decomposition technique. The code is written in the computing language Fortran 2003 and communications are managed by data exchange across adjacent subdomains via the Message Passing Interface (MPI) protocol. The Navier–Stokes solver computes the primary variables of velocity $\mathbf{u }$ and pressure *P* on a fixed and uniform Eulerian mesh by means of Chorin’s projection method (Chorin 1968). Depending on the physical problem, numerical stability requirements and user preferences, the user has a choice of explicit or implicit time integration to either first or second-order. For spatial discretization we use the well-known staggered mesh, MAC method (Harlow and Welch 1965). All spatial derivatives are discretised using standard centred differences, except in the nonlinear term where we use a second-order essentially non-oscillatory (ENO) scheme (Shu and Osher 1989; Sussman et al. 1998). We use a multigrid iterative method for solving the elliptic pressure Poisson equation

8 $$\begin{aligned} \nabla \cdot \left( \frac{1}{\rho } \nabla P \right) = S \end{aligned}$$

where *S* denotes the source term and is a function of the non-projected velocities and interfacial tension. In the case of two-phase flow with large density ratio, the now non-separable Poisson equation is solved for the pressure by a modified multigrid procedure implemented for distributed processors. We have developed a modified parallel 3D V-cycle multigrid solver based on the work of Kwak and Lee (2004). The solver incorporates a parallel multigrid procedure whose restriction and prolongation operators are not associated with each other, contrary to common usage. This method has been successfully implemented to solve 3D elliptic equations where coefficients can be highly discontinuous (Wesseling 1998). The procedure can handle large density discontinuities up to density ratios of $O(10^5)$. The key features of the modified multigrid implementation can be summarized as cell-centered second-order finite-difference approximation of Eq. (8), harmonic approximation of the discontinuous coefficient $1/\rho$, linear interpolation of the residual during the restriction process, cell flux conservation of the error on coarse grids during the prolongation process, parallel Red-Black SOR technique to relax the linear systems on fine grids, and solution of the error using a parallel GMRES algorithm on the coarsest grid. Further details of the code are comprehensively given in Shin et al. (2017).

### Initialisation and boundary conditions

**Fig. 3:**
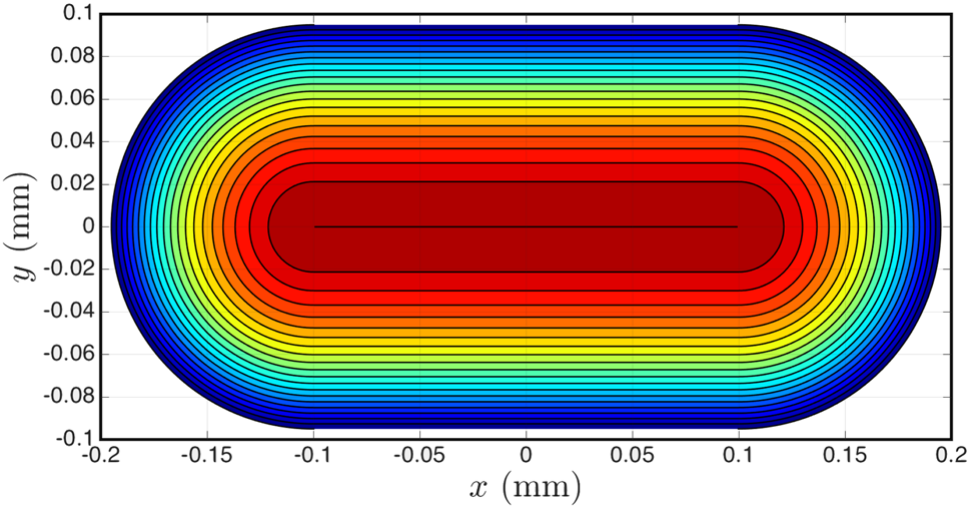
Normalised velocity field at the cross section inlets. The contours are equally spaced with steps of 0.05 and $\left( x_{_0}, y_{_0}\right) = \left( 0,0\right)$

The cross-sectional shape at the entrance of each branch has a shape resembling an oval. This shape, and its dimensions, are highlighted in Fig. 1 (bottom-left) and it is the connection of a rectangular shape ($200\,\upmu \mathrm{m}$ length and $190\,\upmu \mathrm{m}$ width) and two spherical shapes of diameter of $190\,\upmu \hbox {m}$. Despite the complexity of the cross-section, it has the advantage of having a smooth circumference, with no singularities or corners, and so we can set an analytical initial shape for the velocity field at the entrance. The boundary conditions should satisfy the no-slip condition along its circumference and ensure an exact entry flow rate *Q*. Considering the example of a section normal to the *z*-axis (as in Fig. 3), the initialised velocity profile is given by:

9 $$\begin{aligned}&V(x,y)\nonumber \\&\quad =\left\{ \begin{array}{l} \displaystyle {{\mathcal {A}} \left( \frac{(y-y_{_0})^2}{R^2} - 1 \right) \qquad \qquad\qquad\quad\; \;\;\;\,\text{ if } \left| x-x_0 \right| \le L/2}\\ \displaystyle {{\mathcal {A}} \left( \frac{(x-x_{_0} - L/2)^2}{R^2} + \frac{(y-y_{_0})^2}{R^2} - 1 \right) \quad \text{ otherwise }} \end{array}\right. \end{aligned}$$

and

$$\begin{aligned} {\mathcal {A}} = \frac{-Q}{\frac{4}{3} R L + \frac{\pi }{2} R^2}, \end{aligned}$$

where $L=200\,\upmu \hbox {m}$ and $R=95\,\upmu \hbox {m}$ are parameters that define the cross-section and *Q* is the fluid flow rate. Figure 3 highlights the initialised velocity profile at the entrance of a branch for a given flow rate. Here, $x_{_0}$ and $y_{_0}$ refer to coordinate values for the centre position of a branch.

The temporal integration scheme is based on a second-order Gear method (Trucker 2013), with implicit solution of the viscous terms of the velocity components. Each time-step, $\varDelta t$, is chosen for each temporal iteration to satisfy a criterion based on

10 $$\begin{aligned} \varDelta t = \mathrm{min}\left( 0.1 \times \varDelta t_{CFL}, 0.05 \times \varDelta t_{int}, \varDelta t_{\mathrm{cap}} \right) \end{aligned}$$

which ensures stability of the calculations. These bounds are defined by:

11 $$\begin{aligned} \left\{ \begin{array}{l} \varDelta t_{\mathrm{CFL}} \equiv \displaystyle { \min _j \left( \min _{\mathrm{domain}} \left( \frac{\varDelta x_j}{u_j}\right) \right) } \\ \varDelta t_{\mathrm{int}} \equiv \displaystyle { \min _j \left( \min _{\varGamma (t)} \left( \frac{\varDelta x_j}{\Vert \mathbf{V}\Vert }\right) \right) }\\ \varDelta t_{\mathrm{cap}} \equiv \displaystyle { \frac{1}{2}\left( \frac{\left( \rho _{_\mathrm{con.}} + \rho _{_\mathrm{dis.}}\right) \varDelta x_{\min }^3 }{\pi \sigma }\right) ^{1/2}} \end{array}\right. \end{aligned}$$

where $\varDelta x_{\min } = \min _j \left( \varDelta x_j \right)$; Kahouadji et al. (2015) used similar criteria.

**Table 1:** Fluid physical properties for the combinations of liquid–liquid $\textcircled {1}$ and $\textcircled {2}$

	Density $\rho$	Viscosity $\mu$
	($$\hbox {kg/m}^3$$)	(Pa s)
Continuous phase $\textcircled {1}$	855	0.03
Dispersed phase $\textcircled {1}$	1000	0.001
Continuous phase $\textcircled {2}$	920	0.0046
Dispersed phase $\textcircled {2}$	1133	0.006

**Fig. 4:**
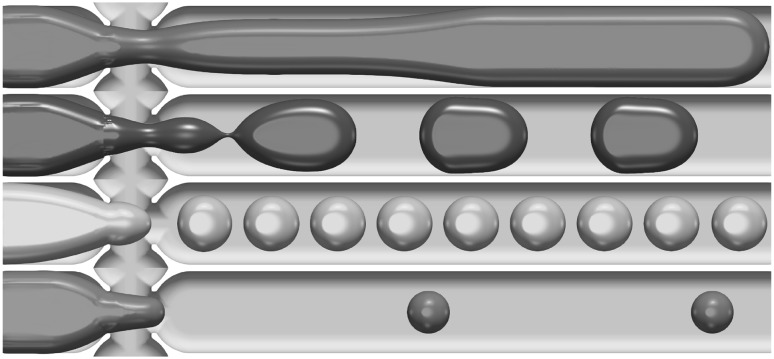
The generic interface shapes showing, from top to bottom, jets, ‘plugs’, ‘pancakes’, and spheres, respectively. The physical properties for both continuous and dispersed phase consists of the combination $\textcircled {2}$ highlighted in Table 1. The flow rate combination between the $\left( \text{ dispersed, } \text{ continuous }\right)$ are from top to bottom (0.07, 0.01), (0.1, 0.05), (0.06, 0.06) and (0.01, 0.08) mL/min, respectively. Experimental snapshots under similar conditions are provided in Fig. 13

## Results

We consider two different liquid combinations $\textcircled {1}$ and $\textcircled {2}$ that correspond to opposite physical situations: in combination $\textcircled {1}$ the dispersed phase is less viscous than the continuous phase, and combination $\textcircled {2}$ has the roles reversed with the dispersed phase more viscous than the continuous one. The corresponding interfacial tensions for these combinations are $\sigma _{_1} = 49$ mN/m and $\sigma _{_2} =29$ mN/m, respectively, and the density and dynamic viscosity values are provided in Table 1. These fluid combinations are then subject to fluid flow rates, and the resulting droplet shapes and flow features are presented.

These fluid combinations, when driven through the junction, have four generic interface shapes for the dispersed phase at the exit branch: (1) spherical drops with a diameter smaller than the cross-junction height ($190\,\upmu \hbox {m}$), (2) ‘pancakes’ resembling a flattened sphere with radius between $190\,\upmu \hbox {m}$ and $390\,\upmu \hbox {m}$, (3) plugs which have an elongated three-dimensional oval shape with length larger than $390\,\upmu \hbox {m}$, and (4) jets where the resulting dispersed phase has the shape of a continuous stratified jet. These generic interface shapes, generated numerically, are shown in Fig. 4 using the fluid combination $\textcircled {2}$ provided in Table 1.

### The dynamics of ‘pancake’ formation

The fluid combination used for this section is $\textcircled {1}$. The typical flow rates are $Q_\mathrm{dis.}=0.05\, \text{ mL/min }$ and $Q_\mathrm{con.}=0.025\, \text{ mL/min }$ in both the top and bottom cross channels; these values are used in all the pancake droplet figures shown. Pancake formation is characterised by periodically spaced identical pancakes that emerge at a fixed frequency. We give the flow rate $Q_\mathrm{con.}$, here and later, as a multiple of the flux in each branch. Breakup in the junction is key to controlling pancake formation, and we begin by inspecting this breakup process.

**Fig. 5:**
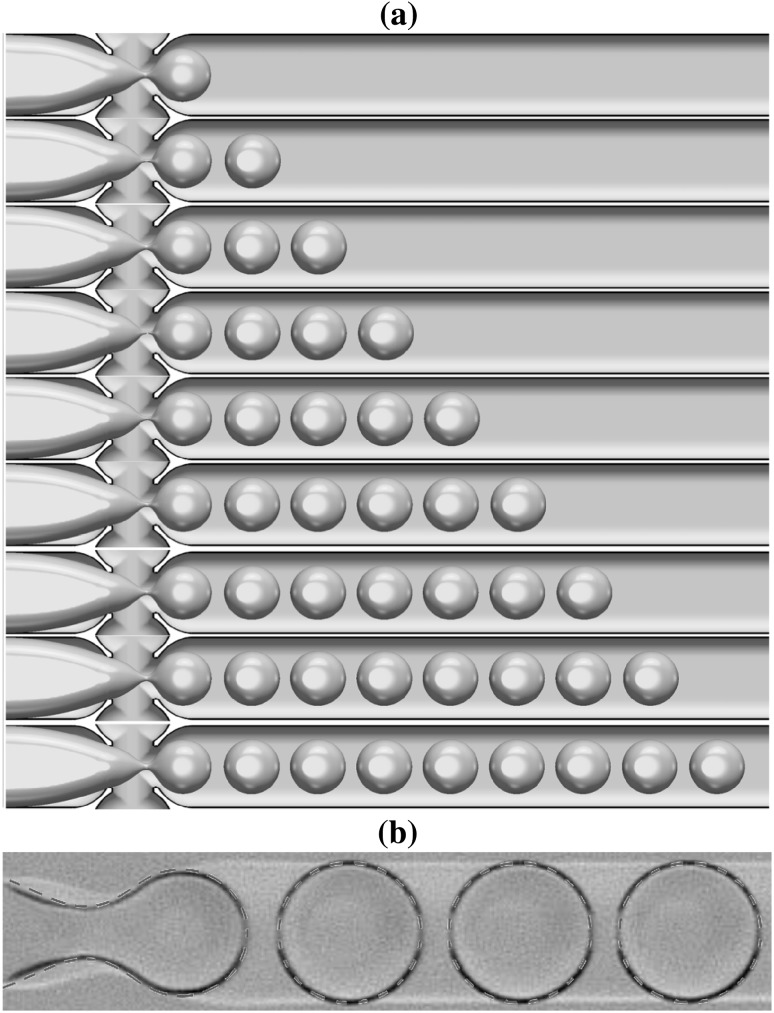
Typical evolution of pancake droplet production for the case of fluid combination $\textcircled {1}$ provided in Table 1 shown in **a**; each pinch-off occurs every $9.3\,\text{ ms }$. **b** A superposition of the interface contour generated numerically and the corresponding experimental snapshot. The dispersed and continuous flow rates are $Q_\mathrm{dis.} = 0.05$ mL/min and $Q_\mathrm{con.} = 2 \times 0.025$ mL/min, respectively

The process is shown in Fig. 5 which depicts the evolution from one droplet to the periodic arrangement; the neck formation at the junction, and pinch-off, are repeated with a precise periodicity of $9.3\,\text{ ms }$. Figure  5 shows sequences of 9 pancakes with the same size $261.3 \,\upmu \mathrm{m}$ and equidistant length of $57.6 \,\upmu \mathrm{m}$. The panel at the bottom of Fig.  5 shows a superposition of experimental and numerical (dashed line) snapshots; there is excellent quantitative agreement between the numerical and experimental results.

**Fig. 6:**
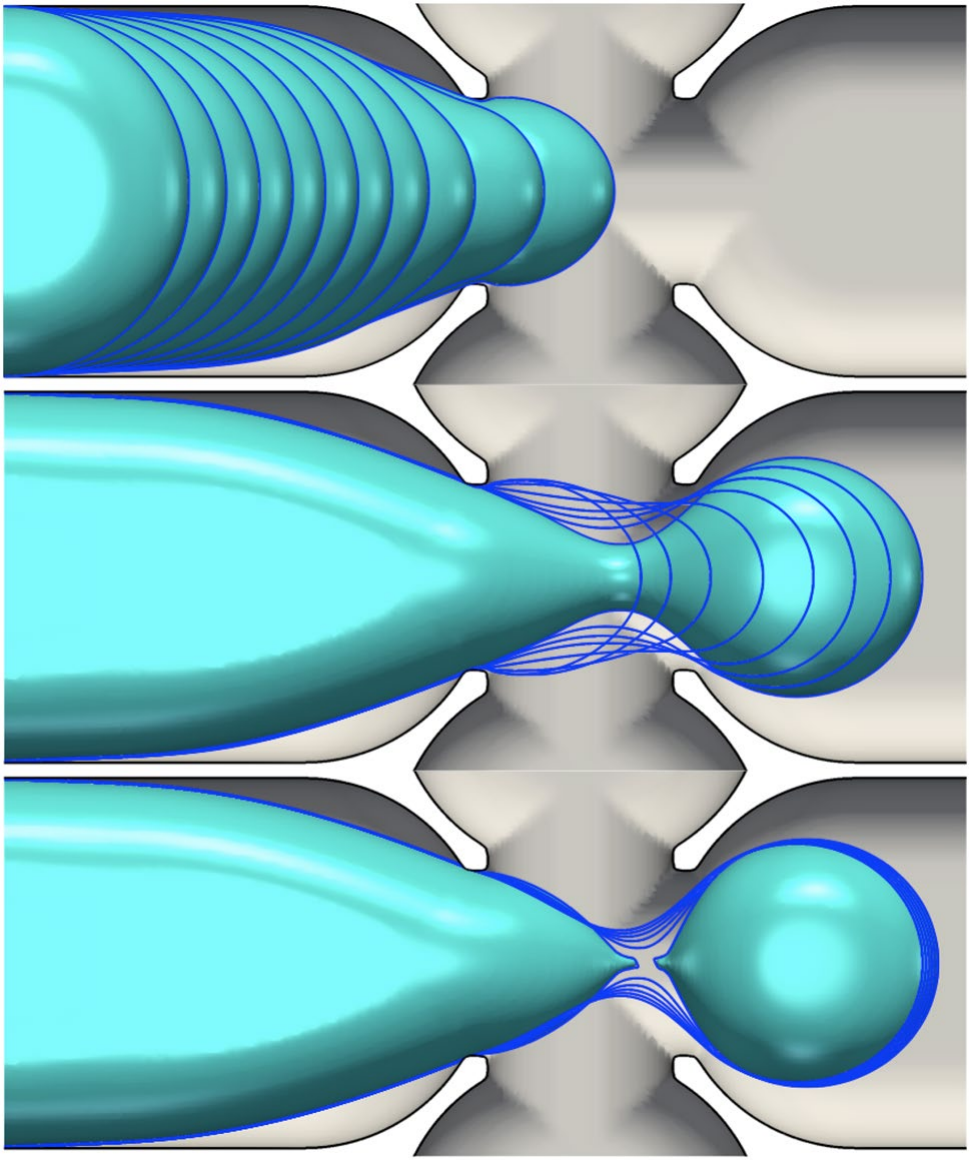
The detail of the neck development in the junction and breakup to form a pancake droplet. Snapshots of the interface position are shown at time intervals of $2.5\,\text{ ms }$, $1.0\,\text{ ms }$, and $0.1\,\text{ ms }$ in the top, middle, and bottom panels, respectively

The detail of neck formation and subsequent breakup are shown in Fig. 6 which highlights the temporal evolution of the interface from its entry into the junction through to forming the first pancake shape in three stages: (1) Fig. 6-top, the interface evolution in the left branch until entering the junction is shown by superposed snapshots separated in time by intervals of $2.5\,\text{ ms }$; (2) once in the junction, Fig.  6-middle, an elongated neck is formed and this process is much more rapid; the snapshot separation here has time intervals of $1.0\,\text{ ms }$. Finally, (3), a very rapid pinchoff in the neck takes place, represented in Fig.  6-bottom, where the time intervals are $0.1\,\text{ ms }$. The numerical simulations show the rapid pinch-off and qualitative behaviour seen in experiments.

**Fig. 7:**
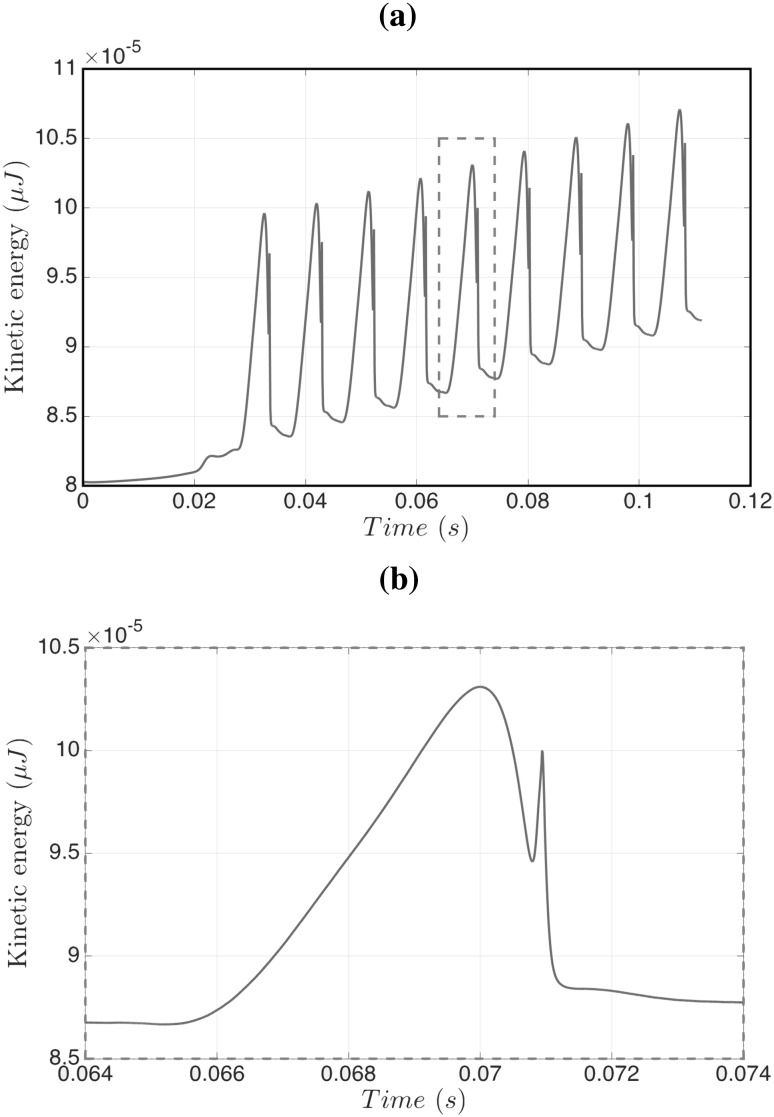
Temporal evolution of the kinetic energy for the formation of the nine pancakes shown in Fig. 5 (**a**), and an enlarged view of the *t* = 0.064–0.074 s interval (**b**)

We now consider the formation and breakup cycle in more detail, a useful diagnostic quantity to consider is the global kinetic energy of the configuration:

12 $$\begin{aligned} E = \iiint \frac{1}{2}\,\rho \,\mathbf{u }^2 \, {\hbox {d}}x {\text{ d}}y {\hbox { d}}z. \end{aligned}$$

**Fig. 8:**
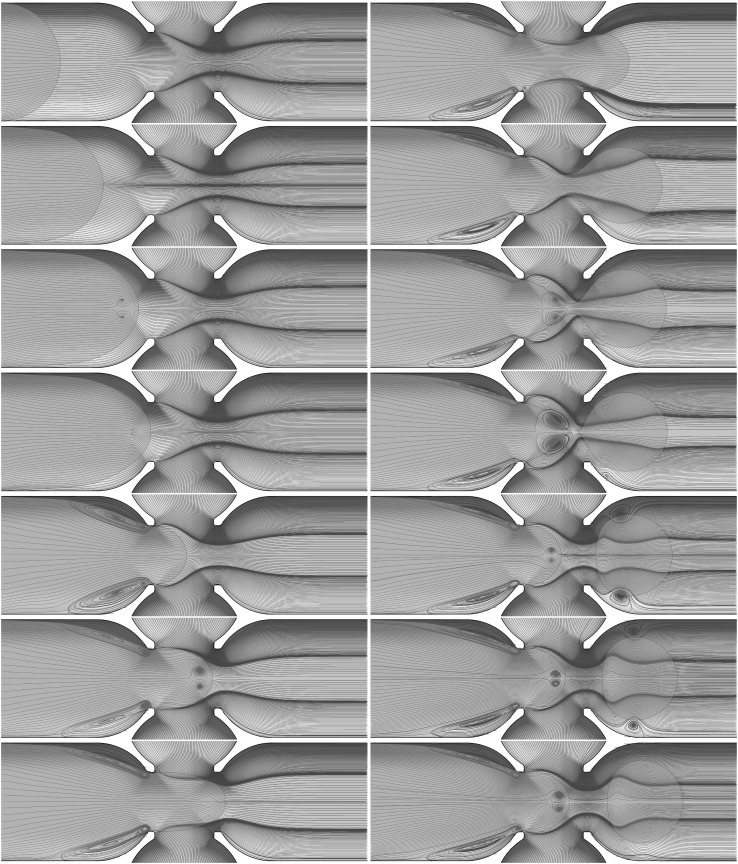
Streamlines highlighting the spatio-temporal evolution of the vortical structures accompanying the formation of pancake droplets for the same parameter values as those used to generate Figs. 5, 6 and 7. Here, $t=0.1,100,180,200,240,270, 280, 300, 330,334, 335,340, 345$, and 350 ms with the panels to be read going down left column and then down the right. An accompanying video is available in the supplementary material

Figure 7a shows this kinetic energy versus time for the pancake formation in Fig. 5, and illustrates the periodic behaviour superimposed on a constant increasing slope, which is due to the density of the dispersed phase ($1000\,\mathrm{kg}/{\rm m}^3$) being larger than the density of the continuous phase ($855\,\mathrm{kg}/{\rm m}^3$). For each cycle, we observe an initial growth of the kinetic energy to a maximum (see Fig. 7-bottom), which corresponds to the neck formation. The kinetic energy then decreases until it jumps rapidly to another local maximum (the sharp peak in Fig. 7b); the peak represents the moment of pinch-off. The pinch-off event is a very rapid process that shows intriguing vortex structures emerging just before breakup and which remain until the neck formation process begins again. Figure 8 shows the streamlines field in both the continuous and dispersed phase highlighting the creation of vortices and their evolution during the pinch-off process.

This vortex motion occurs as, at the moment when the pinch-off occurs, the pancake is pushed forward and simultaneously surface tension forces retract the dispersed phase in the junction backwards. This process generates the formation of a vortex in the dispersed phase about to enter the junction because this retraction is into a flow field that is advancing and pushes the interface forwards again. The vortex remains until the dispersed phase restarts neck formation. The detachment of the pancake drop from the cross-junction also occurs rapidly creating two centrifugal vortices in the continuous phase between the pancake and the wall. From the moment of pinch-off to the final pancake droplet ejection into the exit channel there is a high-velocity field following the pancake droplet. The centrifugal vortices in the continuous phase dissipate rapidly compared to the vortex in the dispersed phase that remains until the next neck formation. This phenomenon is evident in the final snapshot of Fig. 8 and in the accompanying video (supplementary material).

Vortex formation during the pinch-off process in a microfluidic channel was observed experimentally by Chinaud et al. (2015) using two complementary micro-PIV techniques that allows visualisation of the velocity field in both phases. In their experiment this vortex is observed only in the dispersed phase at the edge of the pinch-off location. From the numerical simulations we see that the pair of vortices in the continuous phase has a very low intensity and hence why they were difficult to observe experimentally.

**Fig. 9:**
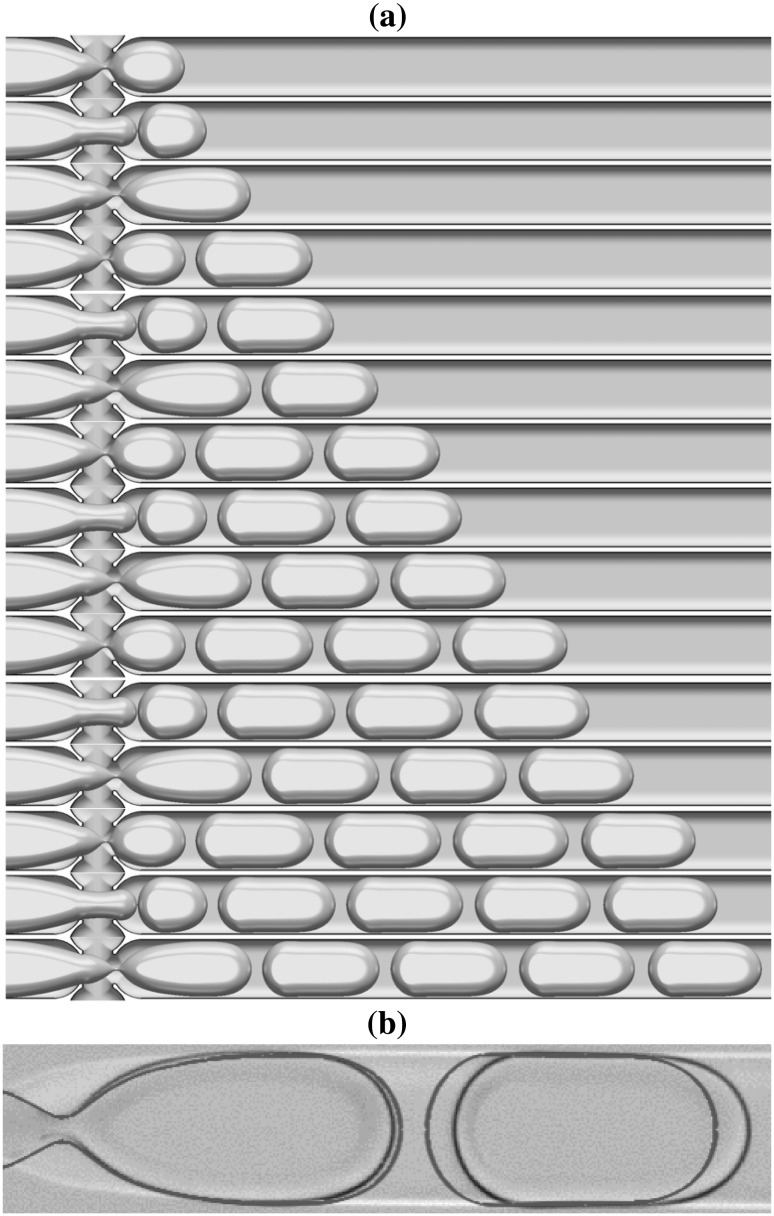
Typical evolution of plug production for the case of fluid combination $\textcircled {1}$ provided in table 1 shown in **a**. The snapshots shown from top to bottom in **a** correspond to $t = 496, 582, 753, 993, 1077, 1249, 1490, 1574, 1746, 1987, 2071, 2243, 2484, 2569$, and 2741 ms, respectively. **b** The superposition of the interface contour generated numerically and the corresponding experimental snapshot. The dispersed and continuous flow rates are fluxes $Q_\mathrm{dis.} = 0.05$ mL/min and $Q_\mathrm{con.} = 2 \times 0.00625$ mL/min

**Fig. 10:**
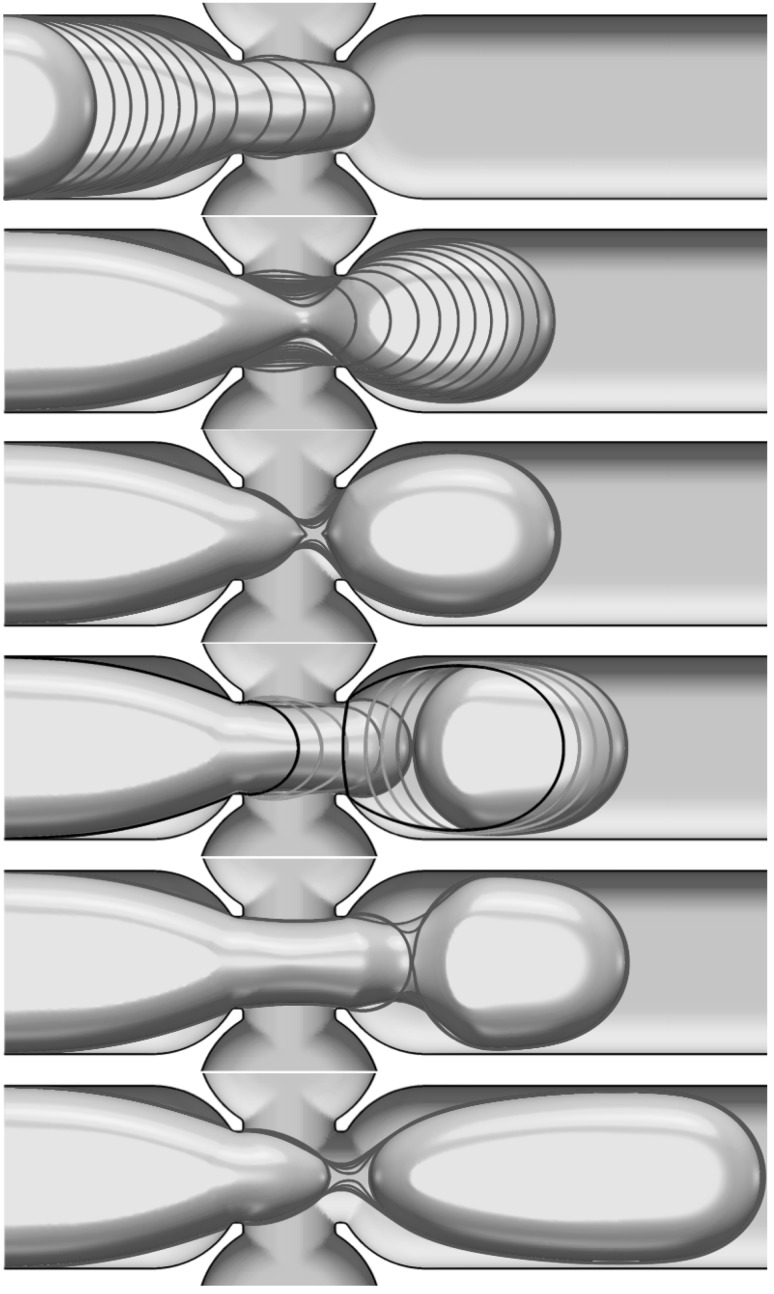
Detailed view of the plug formation process for the same parameters as in Fig. 9. From top to bottom, the snapshots are given at a time period of 2.5, 2, 0.1, 2, 0.1 and 0.1 ms, respectively

### The dynamics of plug formation

**Fig. 11:**
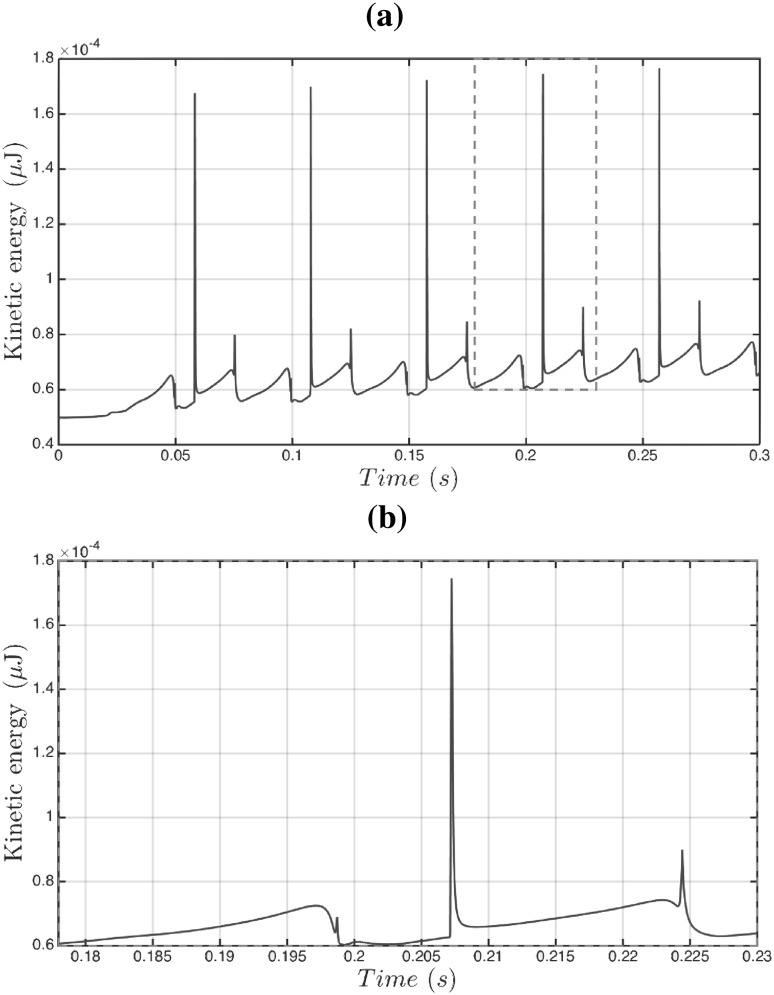
Temporal evolution of the kinetic energy for the plug formation process in Fig. 9 **a**, with an enlarged view of the time interval *t* = 0.18–0.23 s shown in **b**

**Fig. 12:**
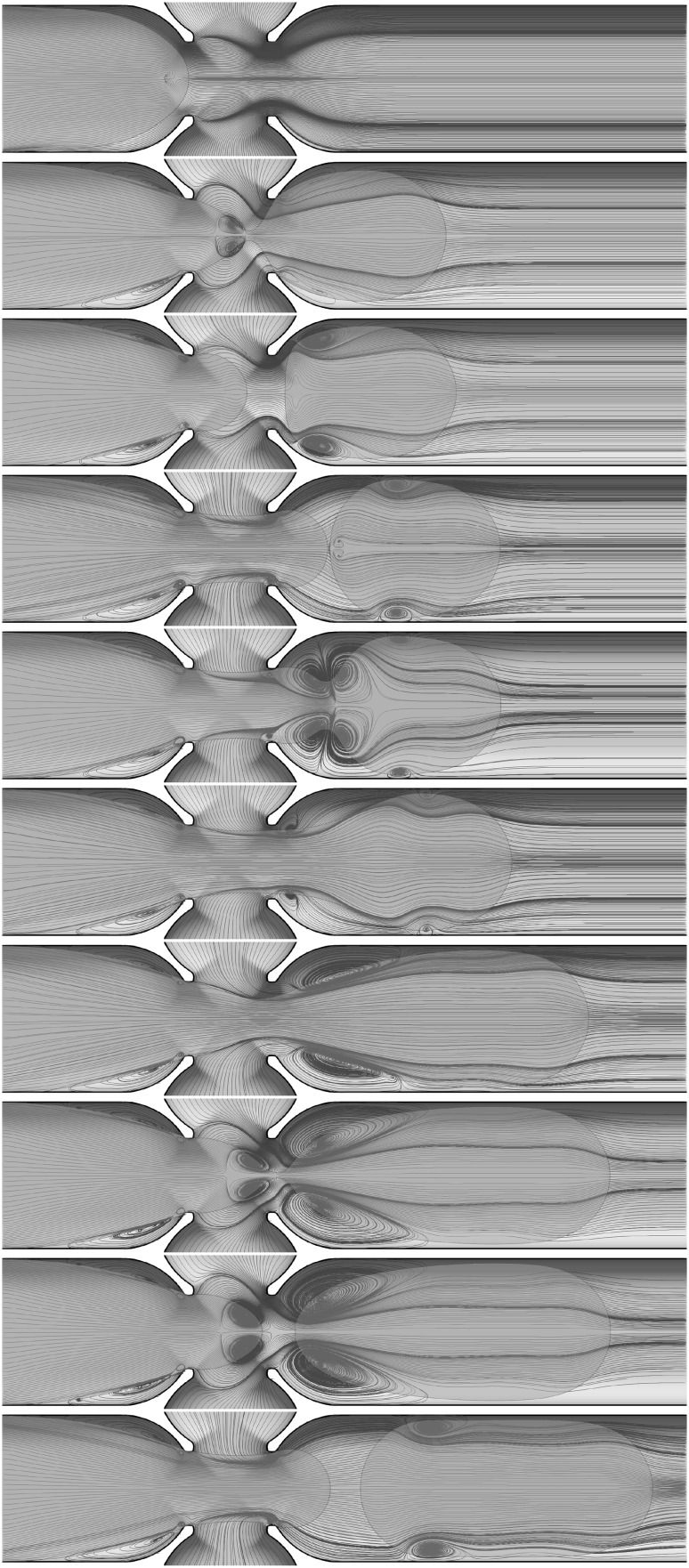
Streamlines highlighting the spatio-temporal evolution of the vortical structures accompanying the formation of plugs for the same parameter values as those used to generate Figs. 9, 10 and 11. Here, $t=200, 496, 512, 582, 583, 600, 720, 753, 754$, and 820 ms for the panels shown from top to bottom, respectively. An accompanying video is available in the supplementary material

The dynamics of the pancake droplet formation shown in the previous section can be summarised as a periodic sequence of pinch-offs. Plug formation is also a result of periodic pinch-offs, with a different time range, but it can be more complex, involving droplet coalescence just after the junction as in the example we choose to illustrate in this section. We keep the same physical properties for both the continuous and dispersed phases and reduce only the continuous-phase flow rate to $Q_\mathrm{con.} = 2 \times 0.00625\,\mathrm{mL}/\mathrm{min}$. The typical sequence of droplets emerging that is observed is shown in Fig. 10. A key difference from pancake droplets is that plug formation involves two pinch-off events and a coalescence event. Figure 9 shows the periodic successions of pinch-off, coalescence and pinch-off process for the formation of five plugs. The time from the first pinch-off to the first coalescence is 8.4 ms., while the coalescence to the second pinch-off that forms a plug took 17.2 ms . From the pinch-off that forms a plug to a pinch-off that provides a new pancake shape for the next plug, the time is 24 ms. Finally, every pinch-off occurs periodically every 49.6 ms and this level of detail is readily extracted from the simulations. All plugs obtained in Fig. 9 are at the same size of $764 \,\upmu \mathrm{m}$ and equidistance of $77 \,\upmu \mathrm{m}$. Figure 10-top shows the details of this plug formation starting from the interface entering the cross-junction with snapshots superposition at constant time of 2.5 ms. As we move down in the figure panels, we see the neck formation and then the first pinch-off (see the second and third panels of Fig. 10). The dispersed phase is now in two parts, the pancake that has emerged from the junction and an interface within the junction; this interface is progressing faster than the pancake drop, and hence it catches up with it and a coalescence event takes place (see Fig. 10-fourth and fifth panels). Finally, another neck forms near the cross-junction leading to the second pinch-off and the plug emerges (see Fig. 10-bottom). To summarise, the plug is formed, by a succession of periodic pinch-off, coalescence, the pinch-off processes. Figure 11 shows the temporal kinetic energy of plug shape formation showing the periodic behaviour with the events clearly separated. Finally, Fig. 12 highlights the complex structure of vortices in both continuous and dispersed phase resulting from the process of two pinch-offs and a coalescence process.

We have chosen to illustrate a computation, in Figs. 9, 10 and 12, corresponding to the most complex situation we encounter: each plug is a consequence of two pinch-off events and a coalescence event. Fig. 9b superposes the experimental snapshot and a numerical contour of the interface; the results have qualitative agreement and also considerable quantitative agreement. The plug size is replicated well, but there is a minor offset between experiment and simulation; this gap does not occur when a plug is the consequence of only a single pinch-off analogous to the pancake formation. The double pinch-off and coalescence is far more sensitive; the results are within experimental error given variability in the experiments in terms of possible minor surface contamination, accuracy of rheological measurements, and experimental data collection.

**Fig. 13:**
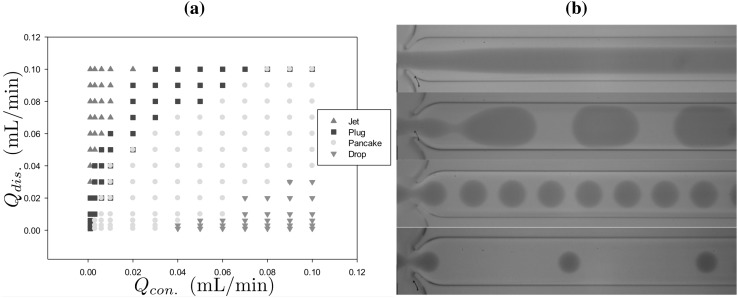
**a** Experimental regime map from Kovalchuk et al. (2018) using the fluid property combinations $\textcircled {2}$ highlighted in Table 1. **b** Experimental snapshots with flow rate combinations similar to Fig. 4 and are from top to bottom (0.07, 0.01), (0.1, 0.05), (0.06, 0.06) and (0.01, 0.08) mL/min respectively

## Concluding remarks

In terms of experiments, in the application we have treated here, regime maps such as that created from the modelling in Fig. 13, are useful predictive tools in experimental work and these can be rapidly created using the modelling process we have outlined in this article. The flow regimes, for this combination of liquids and at the range of flow rates used, are seen to be dominated by the pancake droplet and plugs. The isolated droplets and stable jets are also predicted by the modelling, but for brevity we have omitted them and concentrated upon the more commonly occurring pancake and plug droplets.

Direct numerical simulations (DNS) of the full Navier–Stokes equations have not been utilised before for microfluidics as many solvers have stability issues associated with the interfacial tension. As clearly seen here, front-tracking with the variations that we have developed, overcomes this and hence opens the way towards microfluidic simulations involving, say, reactions, additional physics, surfactant chemistry, phase changes and much more. Such DNS solutions elucidate fine scale features within a microfluidic device junction, such as the vortex creation at pinch-off and the more complex interconnection of events, the pinch-off, coalescence, pinch-off, for plug formation all give valuable insight to the underlying physical processes. Furthermore, the methodology we have allows the geometry of the flow focusing junction to be rapidly redesigned (with minimal computational effort) and that will allow for further precision in terms of fine-tuning the output from such devices. The DNS front-tracking approach is currently being developed as a design tool to aid in the manufacture of microfluidic devices and is being extended to encompass additional physics and chemistry.

Future research avenues for study are to perform numerical simulations featuring three-phase encapsulation, non-Newtonian fluids, and the presence of surfactant as in the recent paper of Shin et al. (2018).

## Electronic supplementary material

Below is the link to the electronic supplementary material.

Supplementary material 1 (AVI 696 mb)

Supplementary material 2 (AVI 995 mb)
